# Supplementary material for: ProteinShader: illustrative rendering of macromolecules
Source: BMC Struct Biol. 2009 Mar 30;9:19. doi: 10.1186/1472-6807-9-19 (PMC2672931; doi:10.1186/1472-6807-9-19)
Supplement: Additional file 1 — ProteinShader program without source code. This compressed file contains the complete ProteinShader program including associated libraries, but no source code. A README.txt file gives an overview of the ProteinShader distribution, and the index.html file in the help subdirectory has directions on getting started with the program as well as a set of tutorials. [file 1472-6807-9-19-S1.zip › ProteinShader-beta-0_9_4-binary/help/index.html]

ProteinShader


# ProteinShader

### *Illustrative Rendering of Macromolecules*


|  |  |  |  |
| --- | --- | --- | --- |
|  |  |  |  |
| Potasium Channel  [PDB:1BL8] | Retinol-Binding Protein  [PDB:1AQB] | Ribonuclease Inhibitor  [PDB:2BNH] | Porin  [PDB:3POR] |

See Tutorials for more images.


## *Art is the lie that helps tell the truth.* - Pablo Picasso


♦ Home


♦ Download


♦ Getting Started

Choose a Link:

Installation

Running

Troubleshooting

PDB Files


♦ Tutorials

Choose a Link:

List of Proteins

Style Options

Saving Images


♦ General Controls

Choose a Link:

Menu Bar

Keyboard Shortcuts

Mouse Motion

Motion Panel

Antialiasing


♦ Cartoon Controls

Choose a Link:

Control Panels

Selection Panel

Radio Buttons

Decorations Panel

Adding Textures

Color Panel

Visibility Panel

Side Chains Panel


♦ Atom Controls

Choose a Link:

Control Panels

Selection Panel

Radio Buttons

Color Panel

Visibility Panel

Scale Panel

Tiling Panel


♦ Developers

Choose a Link:

Compiling

Running

Code Docs

Developer Links


## Overview

By exploiting recent advances in programmable graphics cards, the ProteinShader
program can produce illustrative renderings of proteins that approximate what
an artist might create using pen and ink. The custom texture mapping and
lighting calculations for rendering these images are implemented using vertex
and fragment shaders written in the OpenGL Shading Language, which is supported
on most new graphics cards for ordinary desktop and laptop computers. Vertex
and fragment shaders are also used for mapping text labels and decorative
textures onto the curved surfaces of tubes and ribbons shown in color.

ProteinShader is a free, open-source molecular visualization project distributed
under the GNU General Public License.
To run this platform-independent program a computer must have Java 1.5 or
higher and a graphics card that supports at least OpenGL 2.0 (see the
Getting Started page for details on system
requirements). The most recent version of the program can be obtained from the
ProteinShader Download page on SourceForge, and help on using the program
can be found by using the links and menus in the navigation box on the left
side of this page.

The ProteinShader program knows how to read a
Protein Data Bank
structure file, which is essentially a listing of the xyz-coordinates of
the atoms in a protein. The protein can be displayed in either an atom-style
or a cartoon-style view. An atom-style display uses spheres and cylinders to
represent atoms and bonds, respectively, while a cartoon view uses ribbons or
tubes to represent the backbone of the protein. The appearance of these
displays can be modified extensively with a retractable control panel on
the right side of the canvas, and the
ProteinShader Tutorials use several
example proteins to help familiarize the user with the control panel.

The images on the canvas can be rotated by dragging a mouse across the canvas
or by using a control panel to specify constant rotation. A menu can be used
to save a static image as either a PNG
(Portable Network Graphics)
or JPEG (Joint Photographic Experts Group)
file. To produce publication quality images, an
Antialiasing
control panel can be used to smooth out the rough edges that are often
associated with computer generated images.


## Citing

ProteinShader is *citeware*, so if you use images obtained with
it in a publication or a web page, please cite ProteinsShader as follows:
  
  
 **ProteinShader: illustrative rendering of macromolecules.  
http://proteinshader.sourceforge.net**


## Thesis Document

The ProteinShader program was written by Joe Weber as a Master's thesis
project for the ALM in IT program at the Harvard University Extension School.
The
ProteinShader thesis document
can be downloaded as a 3.9 MB PDF file. The source code included in the
thesis is no longer current, so download the
ProteinShader program and look in the `src`
subdirectory for the most up to date code. Also, there is some minor
degradation of image quality from saving the thesis document as a compressed
PDF, and an antialiasing (image edge smoothing) feature and several other
refinements have been added since the thesis was written.


## Development Notes

For software engineers who want to modify the ProteinShader program, the
Development Notes
page includes directions on how to compile the source code using Ant,
and also gives a brief overview of the ProteinShader program's major
packages. A more detailed description of the program's packages, classes,
and methods is given in the
ProteinShader API, which was generated by using
the Javadoc tool.

A short summary of the most important algorithms used by the ProteinShader
program is given in the next section, and much more detailed expanations can
be found in the
ProteinShader thesis document. A paper on the ProteinShader program will,
hopefully, be published in the near future.


## Illustrative rendering algorithms

To create pen-and-ink style images, the ProteinShader program uses the
real-time halftoning technique of Freudenberg, Masuch, and Strothotte
[1, 2]. Color images
of ribbons and tubes are converted to grayscale using the equation
*gray = 0.30 red + 0.59 green + 0.12 blue*,
and then texture mapping is used to mix a halftone screen (an image file) with
the grayscale lighting calculations by using the smooth threshold function
*color = 1.0 - aliasFactor \* (1.0 - (halftoneColor + grayscaleColor))*,
where *aliasFactor* is a number from 1.0 to 4.0 that influences the degree
of smoothing (antialiasing). To add dark edge lines to the ribbons and tubes,
the ProteinShader uses a combination of lines based on texture coordinates
and lines added to curved surfaces that are nearly perpendicular to the camera.
The intensity of edge lines is calculated using a smoothing function borrowed
from the single-pass wireframe technique of Baerentzen, Nielsen, Gjael, Larsen,
and Christensen [3].

The algorithms for generating three-dimensional tubes and ribbons suitable for
texture mapping are detailed in chapter 4 of the
ProteinShader thesis document. Briefly, the ProteinShader program uses
a combination of Hermite interpolation and SLERP (Spherical Linear
intERPolation) to calculate a spline (a set of piecewise cubic polynomial
equations) and a set of Frenet frames (local xyz-coordinate frames at discrete
points along the spline). The tube or ribbon is produced by sweeping a regular
polygon along the spline, while aligning the polygon to the xy-plane of each
Frenet frame and connecting vertices between polygons at successive positions
along the spline. A repeating pattern of texture coordinates is assigned to
the vertices that define the surface of a tube or ribbon, and these texture
coordinates are then used to map two-dimensional images onto the curved
three-dimensional surfaces. The spline used for generating tubes and ribbons
runs precisely through the amino acid alpha-carbons, so adding balls and sticks
representations of the amino acid side chains creates the appearance that the
side chains are attached to the tubes or ribbons (a spline aligned to
the peptide bonds, rather than the alpha-carbons, would not acheive this visual
effect).


## References

1.
Freudenberg B, Masuch M, Strothotte T:
**Real-time halftoning: A primitive for non-photorealistic shading.**
*Proceedings of the 13th Eurographics workshop on rendering*
2002, 227-231.

2.
Freudenberg B, Masuch M, Strothotte T:
**Real-time halftoning: Fast and simple stylized shading.**
In *Game Programming Gems 4.*
Charles River Media; 2004: 440-443.
http://wwwisg.cs.uni-magdeburg.de/graphik/pub/files/Freudenberg\_2004\_RTH.pdf
, retrieved April 2008.

3.
Baerentzen JA, Nielsen SL, Gjael M, Larsen BD, Christensen NJ:
**Single-pass wireframe rendering.**
*SIGGRAPH Conference Sketches* 2006.
http://www2.imm.dtu.dk/pubdb/views/publication\_details.php?id=4884,
retrieved April 2008.


---

|  |  |  |  |
| --- | --- | --- | --- |
| Hosted by |  |  |  |

Webmaster: Joe Weber,
joe.weber AT alumni.duke.edu
  
Web site last updated: December 30th, 2008
  
© 2007–2008 Joseph R. Weber
